# p63 Is a Promising Marker in the Diagnosis of Unusual Skin Cancer

**DOI:** 10.3390/ijms20225781

**Published:** 2019-11-17

**Authors:** Artem Smirnov, Lucia Anemona, Flavia Novelli, Cristina M. Piro, Margherita Annicchiarico-Petruzzelli, Gerry Melino, Eleonora Candi

**Affiliations:** 1 Department of Experimental Medicine, TOR, University of Rome “Tor Vergata”, 00133 Rome, Italy; 2 Istituto Dermopatico dell’Immacolata-IRCCS, 00163 Rome, Italy; 3 MRC-Toxicology Unit, University of Cambridge, Cambridge CB2 1QP, UK

**Keywords:** p63, p40, skin cancer, squamous cell carcinoma, basal cell carcinoma, Merkel cell carcinoma

## Abstract

Skin cancer is the most common type of cancer worldwide. Ozone depletion and climate changes might cause a further increase in the incidence rate in the future. Although the early detection of skin cancer enables it to be treated successfully, some tumours can evolve and become more aggressive, especially in the case of melanoma. Therefore, good diagnostic and prognostic markers are needed to ensure correct detection and treatment. Transcription factor p63, a member of the p53 family of proteins, plays an essential role in the development of stratified epithelia such as skin. In this paper, we conduct a comprehensive review of p63 expression in different types of skin cancer and discuss its possible use in the diagnosis and prognosis of cutaneous tumours.

## 1. Transcription Factor p63: Gene Structure and Function in Normal Skin and Skin Cancer

p63, like its sibling p73 [1], belongs to the p53 family of transcription factors [2]. Phylogenetic analysis classifies p63 as the most ancient member of the family, followed by p73 and the more recently evolved p53 [3,4]. In evolutionary terms, family members share conserved gene structure and show high sequence homology. Notably, the three members present peculiar functional properties, as indicated, for example, by the phenotypes of the *Trp53, Trp63,* and *Trp73* knockout mice [5,6,7]. Among these, p63 regulates different cellular responses that primarily affect epithelial biology.

### 1.1. Gene Structure and Isoforms of p63

**Figure 1:**
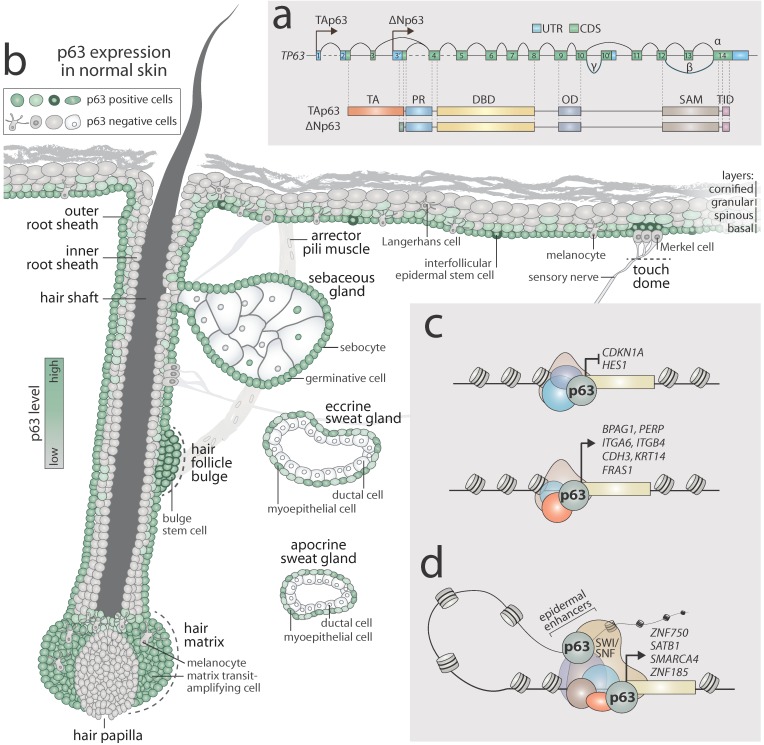
p63 function in normal skin. (**a**) Two different promoters on the *TP63* gene can give rise to TAp63 and ΔNp63 isoforms. These can be further spliced at the C-terminus, producing α, β, or γ isoforms. ΔNp63α is the most abundant isoform in normal skin as well as in skin tumours. p63 protein harbours TA (transcription activation), PR (proline-rich), DBD (DNA-binding), OD (oligomerisation), SAM (sterile α-motif), and TID (transcriptional inhibitory) domains. (**b**) p63 is expressed in most cells of the basal and suprabasal layers of the epidermis but its expression is decreased in the upper spinous layer and absent in the granular and cornified layers of epidermis. p63 intensively marks basal cells of the sebaceous and sweat glands, yet it is not present in mature sebocytes as well as the ductal cells of sweat glands. The cells of the hair matrix, hair bulge stem cells, and outer root sheath show high expression of p63. By contrast, well differentiated cells of the inner root sheath and hair shaft lack p63. Melanocytes and cells of mesenchymal origin, fibroblasts and endothelial cells (not shown), are p63 negative. No data are available regarding p63 expression in Langerhans and Merkel cells (shown as negative); (**c**) In basal layer keratinocytes, p63 plays a crucial role in the maintenance of cell proliferation as well as adhesion. Through direct binding to the promoters of target genes, p63 can repress (upper panel) or activate (lower panel) gene expression; (**d**) During the early stages of keratinocyte differentiation, p63 activates expression of the master regulator of epithelial differentiation, ZNF750, and chromatin remodelers, Brg1 and Satb1. Furthermore, p63 co-operates with chromatin remodeler complex SWI/SNF and binds to epithelial-specific enhancers to allow for the transcriptional activation of a terminal differentiation programme (e.g., ZNF185).

The *TP63* gene gives rise to several protein isoforms resulting from the use of alternative transcription start sites and alternative C-terminus splicing events. Indeed, the *TP63* gene harbours two different promoters that generate two N-terminal isoforms (TAp63 and ΔNp63) (Figure 1a). The TAp63 isoforms present an N-terminus transactivation domain (TA), which is responsible for its transcription activity, while the ΔNp63 isoforms lack this domain and may act as repressors, also exhibiting a dominant-negative effect towards p53 and TAp63/TAp73 [8]. Importantly, ΔNp63 harbours an additional short TA domain, which can positively affect the transcription of specific genes [9]. Through alternative splicing, p63 mRNA generates at least three C-terminal isoforms: p63α, p63β, and p63γ [10], for both the TAp63 and ΔNp63 isoforms. However, unlike p53, the p63α variant presents a protein–protein interaction domain of unknown function, the sterile alpha motif (SAM) [4,11,12], and the transactivation inhibitory domain (TID), which is involved in transcriptional inhibition of the TAp63 isoform [13,14,15] (Figure 1a). 

### 1.2. p63 Expression in Normal Skin

The ∆Np63 isoform is mainly expressed in ectoderm-derived tissues, such as the epidermis, skin appendages, simple epithelia, and the thymus [4,16,17,18,19]. The striking developmental abnormalities found in ΔNp63 genetic-complemented mice [19] and in ∆Np63-null mice [20], demonstrate the indispensable role of the ∆Np63 isoform in epithelial biology. Figure 1b summarises the expression of p63 in normal skin (p63 positive cells are highlighted in green). p63 is expressed in virtually all cells of the basal layer of the epidermis, and the level of its expression decreases towards the outermost terminally differentiated granular and cornified layers. It is detected in all basal germinative cells of the sebaceous gland as well as some sebocytes; however, it is absent in mature excreting cells. In eccrine and apocrine sweat glands, p63 is diffusely expressed in myoepithelial cells but is not present in cells facing the lumen of the ducts. In the hair follicle, p63 can be readily detected in the keratinocytes of the outer root sheath of the hair, stem cells of the hair bulge, and the transit-amplifying cells of the matrix in the bulb of the follicle [21,22]. However, p63 is not expressed in the hair papilla nor in the cells of the inner root sheath. By contrast, neuronal cells and cells of a mesodermal nature, such as the smooth muscle of arrector pili (Figure 1b), dermal fibroblasts, endothelial cells, and adipocytes (not shown in Figure 1b) are p63 negative [23,24,25]. 

Remarkably, no direct evidence has been provided using immunohistochemistry for p63 expression in Langerhans cells and melanocytes. However, p63 was found to be absent in normal melanocytes by polymerase chain reaction (PCR) [26]. This observation was supported by RNA-sequencing performed in melanocytes [27]. Furthermore, no data are available to support p63 expression in the mechanosensory Merkel cells of the touch dome in human skin, even though high levels of p63 have been found in feline Merkel cells [28]. 

In summary, p63 appears to be expressed in stem cells and highly-proliferating transit-amplifying cells of ectodermal origin. However, it is present at low levels or absent in differentiating cells as well as cells of mesenchymal origin. 

### 1.3. Transcription Regulation of Skin Development by p63

As a transcription factor, p63 can activate or repress expression of its target genes through direct binding to their promoter regions (Figure 1c). For instance, in basal keratinocytes, p63 is believed to repress the expression of *CDKN1A* [29], a gene codifying for cyclin-dependent kinase inhibitor 1 (p21), and *HES1* [30], one of the members of the Notch pathway, thus supporting proliferation of transit-amplifying cells and inhibiting their differentiation. By contrast, p63 positively regulates the expression of a plethora of structural proteins to ensure proper cell–cell and cell–matrix adhesion. Among the *bona fide* p63 target genes are components of hemidesmosomes such as *BPAG1* [31] and *PERP* [32]; integrin-coding genes *ITGA6* and *ITGB4* [33]; the P-cadherin-coding gene *CDH3*, a component of adherens junctions [34]; *FRAS1* codifying for an extracellular protein [35]; and *KRT14*, a component of keratin intermediate filaments [19]. Furthermore, p63 has been shown to attenuate the metabolism of keratinocytes [36] by directly activating hexokinase II [37] and cytoglobin [38]. Different chromatin remodelers such as HDAC1/2 [39,40] and KMT2D [41] cooperate with p63 to determine its transcriptional activity. 

Conversely, p63 is closely involved in the process of keratinocyte differentiation in both early and late stages. ZNF750 is one of the earliest transcription factors activated during differentiation by p63 [42]. It induces and later interacts with KLF4 to allow terminal differentiation of keratinocytes [43]. Among the other p63 regulated genes are chromatin remodelers *SATB1* [44] and *SMARCA4* (codifying for Brg1) [45]. Their activity is of pivotal importance in establishing a proper genomic 3D structure and accessibility of the epidermal differentiation complex of genes on chromosome one. 

Recent investigations have unveiled the existence of pre-established epithelia-specific enhancers in undifferentiated keratinocytes. These might interact with the promoter regions of target genes to allow their transcription during differentiation [46]. Notably, p63 was found to cooperate with the Brg1/Brm subunit of the switch/sucrose nonfermenting (SWI/SNF) chromatin remodelling complex on epidermal enhancers [47]. Indeed, it is suggested that p63 bookmarks multiple enhancers already present in proliferating keratinocytes. Many of these loci overlap with active chromatin marks such as H3K27ac. This is the case even if p63 alone cannot induce expression of these genes and needs partners to allow the transcription [48]. One example of this type of network is a component of adherens junctions, ZNF185, whose expression is regulated by p63 during differentiation. Binding to an epithelia-specific enhancer already present in undifferentiated cells, p63 is essential for the maintenance of the enhancer–promoter interaction in order to allow ZNF185 transcription in the suprabasal layers of the epidermis [49]. 

Altogether, p63 is well the established master regulator of epidermal homeostasis and differentiation. Its transcriptional network is an example of an extremely complex spatiotemporal system of positive and negative regulation, which allows for the proper development, self-renewal, and differentiation of the skin. p63 both impedes and triggers keratinocyte differentiation depending on the epithelial compartment, proliferative state, and the network of interacting proteins. 

### 1.4. Transcription Regulation of Skin Cancer Mediated by p63

Although mechanistic aspects of p63 function in the cutaneous basal cell carcinoma (cBCC) and rare skin tumours remain poorly investigated, the role of p63 in tumourigenesis has been intensively studied in squamous cell carcinoma (SCC). A transcriptional programme of p63 in cutaneous SCC is similar to SCC from other epithelial origins such as the head and neck (see recent reviews [50,51]). In the next section, we will highlight specific pathways with a focus on understanding tumourigenic activities of p63 conducted exclusively in the context of cutaneous SCC (cSCC). 

The oncogenic properties of ΔNp63 were established in in vivo experiments that showed overexpression of ΔNp63 enhances mutant Ras-driven tumourigenesis of undifferentiated cSCC [52]. Indeed, p63 contributes to cancer development, preventing oncogene-induced senescence of keratinocytes by activating the expression of the chromatin remodeler LSH [53]. A well-known inhibitor of p53, iASPP, was shown to regulate p63 expression via repression of miR-574 and miR-720 in the normal epidermis [54]. This pathway is epigenetically dysregulated in cSCC, leading to high levels of p63 expression and induction of epithelial-to-mesenchymal transition [55]. However, the nuclear localisation of p63 appears to be an important issue in its oncogenic transcriptional activity. Indeed, Rho-dependent phosphorylation of NUP62 leads to nuclear translocation of p63 and permits its oncogenic activity [56]. Furthermore, p63 can attenuate the function of other members of the p53 family. For instance, p63 represses p73-mediated apoptosis in cSCC [57]. It can also co-operate with mutant p53 in tumour cells to induce the expression of KLF4 and therefore enhance tumour growth [58]. Although the role of p63 in tumour initiation is well established, Bornachea and colleagues reported that, in more advanced tumours, p63 represses the epithelial–mesenchymal transition, thus inhibiting the development of metastases [59]. Overall, these studies indicate that p63 is a multifaceted transcription factor; the ΔNp63 isoform acts as a potent oncogene that exerts its pro-tumourigenic effects by regulating specific transcriptional programmes to sustain malignant cell proliferation and survival. 

## 2. Expression Pattern of p63 in Skin Cancer

**Figure 2:**
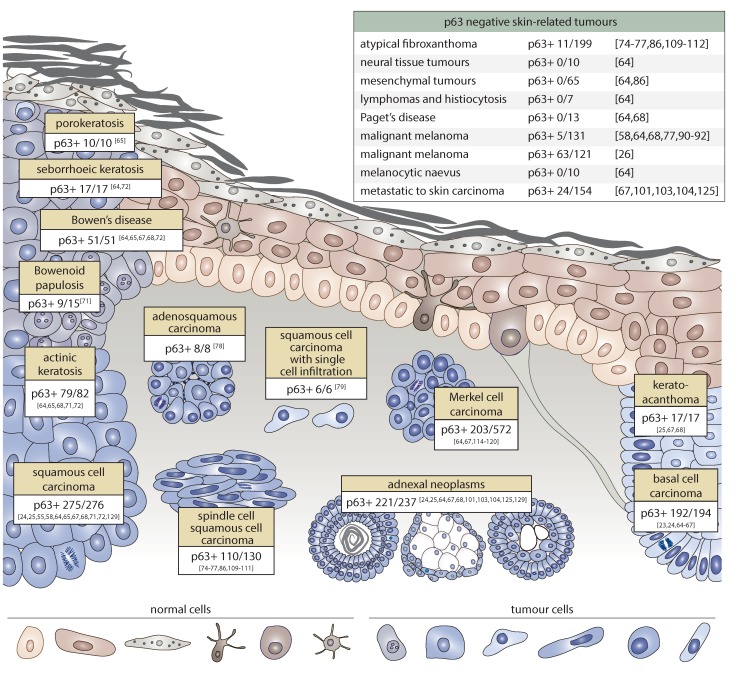
p63 expression in skin cancer. p63 positively marks both benign precancerous lesions (seborrheic-, actinic-, and porokeratoses, keratoacanthoma, Bowen’s disease, and Bowenoid papulosis) and malignant tumours of the skin (basal cell carcinoma, squamous cell carcinoma, spindle cell squamous cell carcinoma (SCC), adenosquamous carcinoma, and SCC with single cell infiltration). Multiple adnexal neoplasms of both a benign and malignant character stain intensively for p63. Merkel cell carcinoma shows positive p63 expression in less than 50% of cases; indeed, its expression is correlated with poor prognosis of Merkel cell carcinoma (MCC). By contrast, neural tissue, mesenchymal tissue tumours, cutaneous lymphomas and histiocytosis, Paget’s disease, and atypical fibroxanthoma lack p63 in most cases. Metastatic carcinoma to skin stains positively for p63 in only 15% of cases. Melanocytic naevi as well as malignant melanoma are virtually all p63 negative, with the exception of one report [26].

Skin cancer is the leading type of cancer worldwide with up to 3 million new cases every year. The most common risk factor for the development of skin cancer is ultraviolet (UV) exposure to the sun. This provokes multiple cytosine-to-thymine transitions that result in DNA mutations [60]. However, in some cases, skin cancer can arise due to other causes such as human papilloma virus infection or chronic arsenic exposure [61]. If detected early and treated appropriately, skin cancer can, in most cases, be cured. However, more aggressive tumours might metastasise and cause death. Therefore, good diagnostic and prognostic markers are needed to ensure the correct diagnosis has been made and the appropriate treatment has been chosen. Herein, we therefore summarise our existing knowledge of p63 expression in different types of skin cancer. A scheme representing the morphological issues of p63 positive tumours as well as a list of p63 negative tumours is presented in Figure 2. 

### 2.1. p63 Expression in Non-Melanoma Skin Cancer

Cutaneous basal cell carcinoma (cBCC) is the most common type of cancer worldwide with almost 750,000 cases detected every year in the USA. cBCC accounts for up to 80% of all non-melanoma skin tumours. Basal cell carcinomas are slowly growing and rarely metastasise. Among the most frequently mutated genes are *TP53* and *PTCH1*. However, cBCC genomes are considered genetically stable compared to those from aggressive types of cancer [62]. Recent reports have indicated that cBCC might arise from stem cells of the hair follicle and touch dome [63]. Multiple studies have demonstrated high expression of p63 in cBCC [23]. Moreover, p63 has been detected in all tumour cells [24,25,64,65,66,67,68]. 

The second most frequent type of skin cancer after cBCC is cutaneous squamous cell carcinoma. With a diagnosis rate of 15–30 per 100,000 people, cSCC accounts for 20% of all skin cancer cases. Notwithstanding a lower frequency with respect to cBCC, cSCC is characterised by a higher incidence of metastases and poorer prognoses [69]. *RAS* and *TP53* are frequently mutated in cSCC. In contrast to cBCC, cSCC can originate from hair follicle bulge stem cells as well as interfollicular epidermal stem cells [70]. p63 is highly expressed in all cSCC [24,25,64,65,67,68,71]. However, unlike cBCC, p63 exhibits a diffuse pattern of expression in cSCC [72] with higher expression on the periphery of the tumour gradually reducing towards the well-differentiated centre [64]. 

A rare variant of cSCC is cutaneous spindle cell squamous cell carcinoma (cSCSCC). The epidemiology of cSCSCC is associated with sun- or radiation-exposure of the skin. Radiation exposure is thought to be correlated with more aggressive phenotypes of the disease. Histologically, cSCSCC is characterised by bizarre and pleomorphic tumour cells that infiltrate deep into the dermis [73]. Several studies have shown that p63 is diffusely expressed in cSCSCC in 70%–100% of cases [74,75,76,77]. Remarkably, two single reports have highlighted the possible utility of p63 in detecting the squamous component in adenosquamous carcinoma of the skin as well as in rare forms of squamous cell carcinoma with single cell infiltration. Indeed, in both cases, the tumours were p63 positive [78,79]. 

Although several cutaneous lesions are of a benign nature, they can be precursors of skin tumours such as cSCC. The most frequent benign tumour of the skin is seborrheic keratosis (SK), which primarily affects people over the age of 50 and has low malignant potential [80]. Another relatively common benign tumour is keratoacanthoma (KA), which arises from the cells of hair follicles [81]. Notwithstanding their benign nature, these pathological conditions show an increased level of p63 positive basaloid cells relative to non-affected skin [64,67,68,72]. Another rare precancerous condition of the skin, generally found in young people, is porokeratosis (PK). It is believed that PK originates from abnormal keratinocytes due to an aberrant programme of terminal differentiation; however, a genetic predisposition has also been demonstrated. PK is mainly benign, although in rare cases it can evolve into skin cancer [82]. In PK, p63 stains positive all cell layers with higher intensity in basaloid cells [65]. Actinic or solar keratosis (AK) is a precancerous condition of the skin with a higher risk of malignant transformation relative to other benign lesions that, if not treated, can evolve into cSCC. In fact, AK is now considered an early form of cSCC in situ [83]. Several research groups have demonstrated high expression of p63 in the basaloid part of skin regions affected by AK [65,68,71,72], although the percentage of p63 positive cells was lower than that of SCC [72]. Bowen’s disease (BD) is cSCC in situ that in 3%–5% of cases leads to invasive and metastatic cSCC [84]. Like other precancerous conditions, BD is characterised by high expression of p63 [64,65,67,68,72]. A very rare form of cSCC in situ is Bowenoid papulosis (BP). This is an uncommon sexually transmitted disease believed to be caused by viruses such as the human papilloma virus [85]. However, it is important to note that p63 positive staining was only found in 9 out of 15 cases [71]. 

Takeuchi and colleagues performed a comprehensive analysis of p63 expression in several skin tumours of different origins, including very rare cases. As expected, p63 was found to be positive in epidermal tumours as well as most adnexal neoplasms (as will be discussed in the next section). In contrast, in all neural tissue tumours, mesenchymal tumours, lymphomas, and histiocytosis, p63 expression was found to be negative [64,86]. Remarkably, extramammary Paget’s disease, a slowly growing rare intraepithelial adenocarcinoma of unknown origin [87], also tested negative for p63 [64,68]. These findings confirm a strong positive correlation between p63 expression and the epidermal origin of neoplastic cells. 

### 2.2. p63 Expression in Melanoma

Although malignant melanoma (MM) is less frequent than cBCC or cSCC, it is among the deadliest forms of skin cancer, leading to 50,000 deaths every year [88]. MM arises from mature melanocytes from both chronically sun-exposed and non-chronically sun-exposed regions of skin [89]. 

No consensus has been reached regarding the pattern of p63 expression in melanoma. Multiple lines of evidence suggest that p63 is virtually never expressed in MM samples (positive in 5 out of 131 samples [64,68,77,90,91,92]) and benign melanocytic naevi [64]. However, Matin and colleagues reported up to 60% of p63 positive specimens (63/121) of melanoma [26]. Furthermore, unexpected evidence recently emerged of a high incidence of mutation in the *TP63* gene in melanoma samples (14.7% of samples) [93]. In contrast to the cancer, mutations within the *TP63* gene are common in epidermal dysplasia [94]. Therefore, the possible impact of *TP63* mutation of melanomagenesis remains elusive. 

## 3. Diagnostic and Prognostic Value of p63 in Skin Cancer 

p63 is used as a gold standard marker for highlighting myoepithelial cells in multiple types of cancer [95,96,97]. In the following section, we discuss the utility of p63 for the diagnosis of different skin tumours and also for prognosis prediction in Merkel cell carcinoma. 

### 3.1. p63 in the Diagnosis of Metastases in Skin

**Figure 3:**
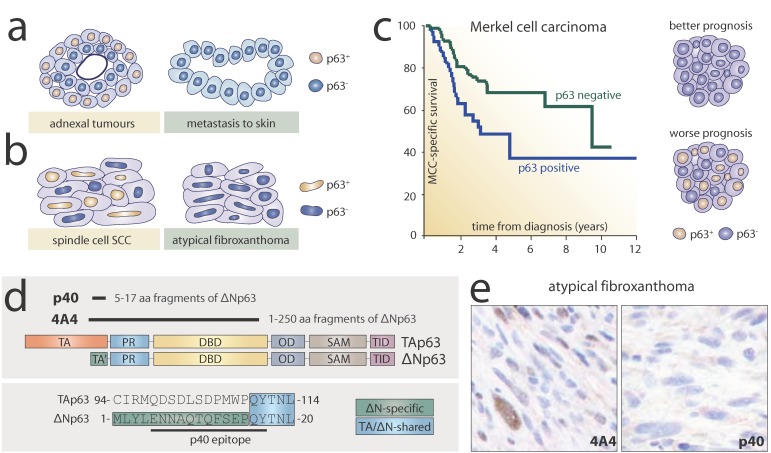
Diagnostic and prognostic value of p63 in skin cancer. p63 can be used in diagnostics to distinguish adnexal tumours from metastases to skin (**a**) as well as spindle cell squamous cell carcinoma from atypical fibroxanthoma (**b**); (**c**) In Merkel cell carcinoma, p63 expression is correlated with poor prognosis. The Kaplan–Meier plot shows the MCC-specific survival of patients with p63-positive and p63-negative tumours (modified from [117]); (**d**) 4A4 antibody is a pan-p63 antibody that recognises the DNA-binding domain of all isoforms of p63. By contrast, the p40 antibody recognises a specific portion of the N-terminus of ΔNp63 isoforms; (**e**) p40 is superior to pan-p63 4A4 in the diagnosis of atypical fibroxanthoma (modified from [111]).

Cutaneous adnexal neoplasms (CANs) are a wide group of benign and malignant skin tumours that normally exhibit differentiation towards one of the cutaneous appendages: hair follicle, sebaceous gland, eccrine, or apocrine glands. Even though the precise origin of these neoplasms remains unknown, it has been hypothesised that CANs might arise from stem cells located within normal cutaneous adnexa [98]. CANs are mainly found in patients aged between 20 and 50 years and most have a benign character [99]. Diagnosis of CANs can be facilitated by histological examination of the morphology of differentiated cells. However, in more complicated cases, specific markers are needed to ensure the correct diagnosis of CANs [100]. As mentioned previously, p63 is expressed in the basal cells of cutaneous appendages in normal skin, therefore, high expression can be expected in adnexal tumours. Indeed, multiple studies have confirmed the presence of p63 in these tumours and proposed the utility of p63 for distinguishing CANs from metastatic carcinoma to skin. Qureshi and colleagues demonstrated strong expression of p63 in cylindroma, poroma, hidradenoma papilliferum, nodular hidradenoma, hidradenocarcinoma, adenoid cystic carcinoma, sebaceous carcinoma, porocarcinoma, digital papillary adenocarcinoma, and syringomatous carcinoma. By contrast, 92% of metastatic carcinoma samples of different origins were found to be negative for p63, except for metastasis of oesophageal and urothelial carcinoma [101]. The same group later reported that p63 can be useful in recognising primary cutaneous mucinous carcinoma from those that metastasise to other tissues by highlighting p63 and CK5/6 positive myoepithelial cells (Figure 3a) [102]. Other reports have confirmed high p63 expression not only in primary cutaneous adnexal carcinoma but also in their cognate metastases (eccrine, apocrine, pilomatrical, sebaceous, and hidradenocarcinoma). Moreover, in the same study, 20 out of 20 metastases analysed in skin from other tissues tested negative for p63 [103,104]. Recent reports have strongly supported previous observations demonstrating high specificity (100%) and sensitivity (58%) in distinguishing CANs from metastasis of breast cancer to skin [105]. Another challenge for pathologists can be well-differentiated neuroendocrine tumours (WDNT) that originate in the stomach or lungs and can metastasise to skin. A total of 10 out of 10 samples of WDNT tested negative for p63, which can be useful in distinguishing them from CANs [106]. 

### 3.2. p63 in the Diagnosis of Atypical Fibroxanthoma

Atypical fibroxanthoma (AFX) is a rare benign tumour with a favourable prognosis that mainly occurs in sun-exposed skin on the head and neck regions of elderly patients, predominantly men. AFX is believed to be of fibroblastic origin and is characterised by myofibroblastic differentiation. The diagnosis of AFX is difficult and is performed by excluding other malignancies such as cSCSCC [107,108]. 

p63 was found to be negative in 188 out of 199 samples. Notably, in several reports the expression of p63 in AFX samples was assessed in parallel with its expression in cSCSCC. Indeed, 110 out of 130 cSCSCC samples stained positively for p63 [74,75,76,77,86,109,110,111,112]. Thus, in addition to the classical panel of cytokeratins, p63 is suggested for use in excluding cSCSCC in AFX diagnosis (Figure 3b). 

### 3.3. p63 in the Prognosis of Merkel Cell Carcinoma

Merkel cell carcinoma (MCC) displays neuroendocrine features and is a rare but aggressive type of skin cancer. It mainly affects elderly patients (>75 years old) and can be caused by chronic UV exposure or Merkel cell polyomavirus. The origin of MCC remains elusive; however, it is speculated that this kind of cancer may arise from Merkel cell precursors as well as pre-B cells or fibroblasts, rather than mature post-mitotic Merkel cells. MCC is characterised by a high risk of metastases and death [113]. Due to the rare character of MCC, initial investigations based on small samples showed negative staining for p63 in MCC; indeed, p63 was proposed as a differential marker for distinguishing MCC from other epithelial types of cancer [64,67]. However, Asioli and colleagues published a striking report in 2007 demonstrating that more than 50% of MCC samples stained positively for p63. Furthermore, p63 expression was correlated with a poorer prognosis [114]. This finding was supported further by the same group [115] as well as by others (Figure 3c) [116,117,118]. Notwithstanding this clear correlation between p63 status and survival, Lim et al. reported that only 5 out of 103 samples positively stained for p63 [119]. Another group later found similar results [120]. One possible explanation for this may be the geographical factor: Higher percentages of p63 positive cases (up to 50%) were reported by American, Canadian, and European groups, and lower percentages of cases (5%–17%) by Australian groups. Therefore, the clinical utility of p63 as a prognostic marker remains uncertain. Nevertheless, the unusual differential pattern of p63 positivity raises many questions about the role of p63 role in MCC tumourigenesis. For instance, Asioli and colleagues detected TAp63, ΔNp63, as well as β isoforms by PCR in MCC tumours. It is speculated that p63 may be expressed in a portion of tumour cells, triggering the switch towards stemness and thus rendering the tumour more aggressive [115].

### 3.4. p40 as an Alternative to the Pan-p63 Antibody

Since the discovery of p63 [4], researchers have been interested in understanding the role and patterns of expression of multiple isoforms of p63 in normal tissues as well as in cancer. Indeed, as early as 2001, Senoo and colleagues investigated by PCR the expression of different isoforms of p63 in SCC and concluded that Δp73L, now known as ΔNp63α, is the most abundant isoform of p63 in squamous cell carcinoma of the skin [121]. Two years later, Geddert and colleagues used a ΔNp63 specific antibody (p40) for the analysis of oesophageal SCC, which indeed recapitulated the staining using the pan-p63 antibody 4A4 [122]. The position of epitopes recognised by pan-p63 and p40 is shown in Figure 3d. However, it was only in the 2010s that the p40 antibody was re-introduced into diagnostics as a more precise molecular marker for squamous carcinoma. Bishop and colleagues reported an equal sensitivity of p40 with respect to pan-p63 in lung SCC, but higher specificity in lung adenocarcinomas and large cell lymphomas [123]. 

The reason for the discrepancy between 4A4 and p40 staining patterns remains unclear. One possible explanation might be that tumour cells express not only ΔN but also TA isoforms of p63 that are recognised by 4A4 but not p40. Another, more plausible, hypothesis is that different staining results are due to the non-specific binding of 4A4 to other members of the p53 family or even other proteins. Indeed, it has been shown that the 4A4 antibody can readily recognise p73 isoforms in vitro [124]. 

Several reports have also confirmed the utility of p40 in the diagnosis of skin tumours. Lee et al. demonstrated higher specificity of p40 with respect to pan-p63 (92% vs. 83%, respectively) in distinguishing adnexal carcinoma from metastases in skin [125]. As described previously, atypical fibroxanthoma can be a challenge for diagnosis. Notably, Henderson and colleagues reported the usefulness of p40 in distinguishing AFX from cSCSCC: 8 out of 27 cases of AFX were variably positive for pan-p63 whereas none were positive for p40, rendering p40 superior to pan-p63 in AFX diagnosis (Figure 3e) [111]. These findings were further supported by another study [112]. Ha Lan and colleagues debated the superior quality of p40; while p40 and pan-p63 antibodies gave similar results for AFX and desmoplastic melanoma, pan-p63 showed higher sensitivity in the detection of cSCSCC relative to p40 (81% vs. 56%, respectively) [86]. Notwithstanding this discrepancy, other recent studies have suggested using p40 in the diagnosis of skin cancer [126,127] and as a gold standard for other tumours as non-small cell lung cancer [128]. In line with increased evidence for the usefulness of p40, a monoclonal MMp40 antibody against ΔNp63 (clone BC28) was produced and is currently commercially available for diagnostics (Roche, Cat No. 790-4950). The high specificity and sensitivity of MMp40 BC28 was confirmed in multiple types of cancer such as lung, prostate, breast, and skin cancer [129].

## 4. Concluding Remarks 

**Figure 4:**
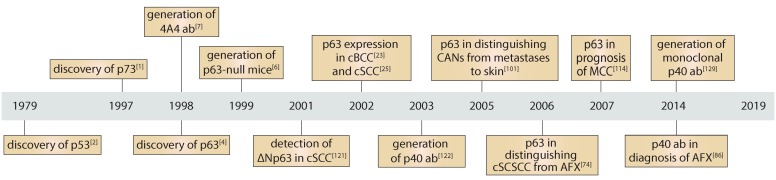
Timeline of important discoveries in the study of p63 in skin cancer. Since the discovery of p63 in 1998, significant progress has been achieved in understanding its role in normal epithelia as well as in cancer. In the early 2000s, multiple lines of evidence demonstrated its possible use as a diagnostic marker in excluding cutaneous spindle cell SCC (cSCSCC) and adnexal neoplasms during the diagnosis of atypical fibroxanthoma and metastases to skin, respectively. Development of the ΔNp63-specific antibody, p40 (ab), improved the specificity of diagnosis. Moreover, p63 expression is associated with a poorer prognosis in Merkel cell carcinoma, rendering it a new predictive marker of MCC.

Forty years ago, the master tumour suppressor p53 was discovered, heralding a new era of understanding regarding the ubiquitous molecular mechanisms of cellular defence against cancer initiation and progression (Figure 4). Almost two decades later, p73 and p63 were described as close homologues of p53. Further investigation led by the research group of Frank McKeon unveiled the essential role of p63 in epithelial development and homeostasis. Multiple lines of evidence supported the existence of the N-truncated isoform, ΔNp63, predominantly expressed in stratified epithelia.

Recent studies carried out with primary keratinocytes have elucidated a very complex and thorough transcriptional programme governed by p63. Via interaction with chromatin remodelers, p63 enables the establishment of a well-organised three-dimensional genomic architecture to ensure the proper development of the skin as well as its homeostasis. Not surprisingly, in skin cancer, p63 pathways can be altered, disrupting the balance between proliferation and differentiation.

The paramount importance of p63 in epithelia development encouraged researchers and pathologists to explore its expression in epithelial cancer. Multiple groups in the early 2000s carried out an extremely comprehensive study of different types of skin cancer using immunohistochemistry, providing strong evidence for high expression of p63 in virtually all types of non-melanoma skin cancer, including pre-cancerous lesions as well as rare forms of squamous cell carcinoma. By contrast, several skin-related tumours of non-epithelial origin such as atypical fibroxanthoma, along with diverse metastases to skin from other organs, are in most cases p63 negative. 

Even though no decision has been made regarding the use of p63 as a gold standard in skin cancer, multiple reports have proposed its utility in differential diagnosis. Furthermore, p63 has emerged as a putative marker for outcomes in Merkel cell carcinoma, where high p63 expression is associated with a poorer prognosis. Based on the pre-clinical studies, summarised in this review, we can speculate that p63 might be an important and targetable molecule, although no clinical trials have been reported that address how the level of p63 expression could modify current therapies. Nevertheless, a growing body of evidence suggests that p63 could be a promising diagnostic and predictive marker for unusual cases of skin cancer.

## Abbreviations

AFX	atypical fibroxanthoma
AK	actinic keratosis
BD	Bowen’s disease
BP	Bowenoid papulosis
CANs	cutaneous adnexal neoplasms
cBCC	cutaneous basal cell carcinoma
CK	cytokeratin
cSCC	cutaneous squamous cell carcinoma
cSCSCC	cutaneous spindle cell SCC
KA	keratoacanthoma
MCC	Merkel cell carcinoma
MM	malignant melanoma
PCR	polymerase chain reaction
PK	porokeratosis
SK	seborrhoeic keratosis
UV	ultraviolet
